# Membrane Deformation and Its Effects on Flow and Mass Transfer in the Electromembrane Processes

**DOI:** 10.3390/ijms20081840

**Published:** 2019-04-13

**Authors:** Giuseppe Battaglia, Luigi Gurreri, Girolama Airò Farulla, Andrea Cipollina, Antonina Pirrotta, Giorgio Micale, Michele Ciofalo

**Affiliations:** Dipartimento di Ingegneria, Università degli Studi di Palermo, viale delle Scienze Ed. 6, 90128 Palermo, Italy; giuseppe.battaglia03@unipa.it (G.B.); girolama.airofarulla@unipa.it (G.A.F.); andrea.cipollina@unipa.it (A.C.); antonina.pirrotta@unipa.it (A.P.); giorgiod.maria.micale@unipa.it (G.M.); michele.ciofalo@unipa.it (M.C.)

**Keywords:** electrodialysis, reverse electrodialysis, ion exchange membrane, profiled membrane, CFD, pressure drop, mass transfer, structural mechanics, fluid-structure interaction

## Abstract

In the membrane processes, a trans-membrane pressure (TMP) may arise due to design features or operating conditions. In most applications, stacks for electrodialysis (ED) or reverse electrodialysis (RED) operate at low TMP (<0.1 bar); however, large stacks with non-parallel flow patterns and/or asymmetric configurations can exhibit higher TMP values, causing membrane deformations and changes in fluid dynamics and transport phenomena. In this work, integrated mechanical and fluid dynamics simulations were performed to investigate the TMP effects on deformation, flow and mass transfer for a profiled membrane-fluid channel system with geometrical and mechanical features and fluid velocities representative of ED/RED conditions. First, a conservatively high value of TMP was assumed, and mechanical simulations were conducted to identify the geometry with the largest pitch to height ratio still able to bear this load without exhibiting a contact between opposite membranes. The selected geometry was then investigated under expansion and compression conditions in a TMP range encompassing most practical applications. Finally, friction and mass transfer coefficients in the deformed channel were predicted by computational fluid dynamics. Significant effects of membrane deformation were observed: friction and mass transfer coefficients increased in the compressed channel, while they decreased (though to a lesser extent) in the expanded channel.

## 1. Introduction

Processes based on ion exchange membranes are increasingly being adopted in industrial applications, from water treatment [1] to food processing [2] and energy harvesting [3], as both environmentally friendly and economically attractive. In electrodialysis (ED) [4], ions are driven by an imposed electric field from a dilute electrolyte solution to a concentrate one. Conversely, reverse electrodialysis (RED) [5] harvests electrical energy from the controlled mixing of two solutions at different salt concentration. ED and RED units are built by alternately stacking anion- and cation- exchange membranes, separated by net spacers or built-in profiles creating the fluid channels where the two solutions (concentrate and diluate) flow. The two membranes and the two solutions form the repeating unit, referred to as cell pair. Spacers cover part of the membrane surface, thus reducing the actual active area, and increase the electrical resistance, as they are electrically non-conductive.

Profiled membranes have recently been presented as an innovative solution to overcome net spacers drawbacks [4,6,7]. Profiled membranes simplify the stack assembly avoiding the use of spacers, and may improve the process performance. Numerical simulations [8,9,10] and experimental lab scale tests [4,11,12,13,14,15] have confirmed their potential benefits. However, the actual performance of profiled membranes stacks depends on the specific profile geometry. Simple geometries (e.g., pillar or ridges profiles) are characterised by reduced hydraulic friction, but may exhibit lesser mixing properties than spacers [10,12,13,14,16]; on the other hand, improved profile shapes may provide better trade-off solutions among pressure drops, mixing and Ohmic resistance, thus improving the stack performance [8,15,16].

In membrane-based processes, a trans-membrane pressure (TMP) between the different solutions flowing through a module may be a design feature or may arise for various reasons (e.g., flow arrangement or differences in geometry, flow rate or physical properties). This may lead to local deformations of membranes and membrane-bounded channels. As a result, the channel geometry (shape and average size) may be modified with respect to the nominal one, affecting fluid dynamics and transport mechanisms (of mass, heat, ions) and, thus, the process performance.

The effects of membrane/channel deformation have been studied in the context of different processes. She et al. [17] tested pressure retarded osmosis (PRO) modules at pressures up to 16 bar. Experimental performance became worse than theoretical predictions as the hydrostatic pressure increased; this difference was attributed to a more severe membrane deformation at high pressures. Later, She et al. [18] studied in detail the influence of spacer geometry on PRO efficiency under pressure loads up to 20 bar. The spacer with the largest mesh pitch gave the poorest performance in terms both of power density and of pressure drop.

Karabelas et al. [19] investigated the influence of the compressive stresses that arise in reverse osmosis (RO) spiral wound membrane modules, provided with spacers, during the assembly stage. The stresses localized at the membrane-spacer contact regions were systematically addressed as functions of spacer compaction, channel gap, membrane indentations and pressure drop. Interestingly, mild applied pressures (1–2 bar) were sufficient to cause significant effects. Correlations for the frictional losses were obtained for various applied pressures and were implemented into a process model predicting the performance of RO units.

Huang [20] simulated flow and heat transfer in deformed channels for liquid-to-air membrane energy exchanger (LAMEE) units. Membrane deformation was not actually computed, and the deformed membrane was modelled as a spherical surface. As membrane deformation increased, the friction coefficient was found to increase in the compressed (air) channel and to decrease in the expanded (liquid) channel. Heat transfer was affected by deformation in a complex way.

The influence of channel deformation on the performance of proton exchange membrane fuel cells (PEMFC) was assessed in several studies following similar approaches. Shi and Wang [21] predicted the compression of the porous gas diffusion layer due to the clamping (assembly) force, and simulated fluid dynamics, mass transport and electrochemical phenomena in the deformed geometries. The authors considered a serpentine channel and found that the assembly compression of the units enhanced pressure drop in the fluid channels, and that the process performance was particularly affected by deformation at high current densities. Zhou et al. [22] simulated a unit with a single straight channel including the membrane. As expected, most of the deformation was found to occur in the porous gas diffusion layer due to its lower mechanical stiffness. The spatial distributions of porosity and permeability were computed and the effects of assembly pressure, gas diffusion layer thickness and membrane features were assessed.

Hereijgers et al. [23] measured membrane deflection and mass transfer coefficients in membrane microcontactors using round and diamond-shaped pillar spacers of different pitch. They found that trans-membrane pressure exhibited a minimum as the spacer pitch was made to vary, and that membrane deflection had a positive or negative impact on mass transfer depending on the diffusion coefficients in the two immiscible phases.

Time-dependent membrane deformation has recently been considered as a possible means to improve process performance. Moreno et al. [24] introduced the concept of “breathing cell” for reverse electrodialysis systems. In the breathing cell, the channels thickness changes dynamically due to the intermittent (5–15 cycles per minute) closure of an outlet valve in the concentrate channels. As a result, the Ohmic resistance of the diluate compartment (which is the predominant one) decreases. Some effects on concentration polarization are also expected. This cyclic operation was shown to yield higher net power densities in a range of flow rates.

Some ED/RED practical applications are poorly affected by these issues (TMP ≈ 0). However, in prototype and industrial size stacks with non-parallel flow layouts (cross flow, counter flow) and/or with asymmetric channels (different geometries, fluid properties, flow rates), where the pressure distribution in the two compartments is different, appreciable values of TMP may arise. In particular, when some factors enhancing pressure drop are present, TMP values amounting to some tenths of a bar can be exhibited (higher TMP levels can cause severe risks of leakages [25,26,27]).

For example, in the cross-flow RED prototype units (44 × 44 cm^2^) installed within the REAPower project [28], pressure drops from ~0.2 to ~0.9 bar were measured at flow velocities up to 1 cm/s [29]. Despite some of the pressure drop can be supposed to occur in the manifolds, a significant part of it is expected to occur in the channels, thus causing the onset of non-negligible TMP values. Moreover, the compartments were asymmetric, because the viscosity of the concentrated solution (brine) was almost twice that of the dilute feed, thus causing an unbalanced pressure distribution in the two solutions. Larger *TMP* values (up to ~1.5 bar) were measured by Hong et al. [27] in a cross-flow RED stack (35.5 × 35.5 cm^2^) fed with inlet velocities up to ~5 cm/s, which provided a significantly lower electrical power (less than half) compared to an equivalent parallel-flow stack. Although the authors attributed this decline in performance to issues of internal leakage, an important effect of deformation can be supposed.

ED units operate with fluid velocities higher than those typical of RED (in order to increase the limiting current density) and, despite the usually higher channel thickness, exhibit large pressure drops [1]. For example, Wright et al. [30] performed ED tests in a bench-scale unit and in a commercial-scale unit with parallel flow, measuring pressure drops up to ~0.65 bar and ~1.30 bar, respectively, at fluid velocities up to ~9 cm/s. If such operating conditions were adopted in non-parallel flow arrangements, they would lead to significant levels of *TMP*.

Recent studies showed that asymmetric channels are optimal for RED applications [31,32]. However, they can be affected by *TMP*-related issues. For example, in ref [32] it was shown that for the couple of NaCl solutions 15–500 mol/m^3^ fed with parallel flow in a stack 50 cm long, the optimum thickness and fluid velocity are ~400 μm and ~1.4 cm/s for the concentrate and ~217 μm and ~2.6 cm/s for the diluate. The pressure drop predicted by Computational Fluid Dynamics (CFD) correlations is 0.07 bar for the concentrate and 0.46 bar for the diluate, thus giving a maximum TMP located at the inlet equal to ~0.39 bar.

It must also be added that ion exchange membranes may have very different mechanical features. The Young modulus (*E*) may vary within a broad range from 10 MPa to 1 GPa [33,34,35,36,37,38,39,40,41,42,43] or even to higher values in some cases [44,45], but decreases with ageing due to membrane usage [34,35,36,44]. Moreover, the new generation membranes are manufactured with low thickness, e.g., from 80 to 250 µm [46,47]; even lower values can be found among commercial membranes and experimental membranes prepared in laboratory [48]. A theoretical study [49] has recently found optimal thicknesses of 15–20 and 50–70 µm for ED and RED applications, respectively. Therefore, it is quite common that ion exchange membranes exhibit a low stiffness, due to the combined effects of a low *E* and a low thickness. This feature makes the membranes susceptible to large deformations in stacks with a non-negligible TMP, depending also on the spacer features.

In particular, a fluid-membrane mechanical interaction will be triggered, which will find an equilibrium state characterized by some distribution of pressure, geometry, flow rate, hydraulic friction, mass transfer coefficient, current density, Ohmic and non-Ohmic resistances in both compartments. Compared to the nominal conditions, the values of any of the above quantities under deformed conditions may be: (i) either higher or lower in the whole channel (e.g., in asymmetric configurations); (ii) higher in some parts of the channel and lower in other ones (e.g., in non-parallel flow arrangements). In both cases, these deviations from the undeformed conditions may impair the process performance due to the lack of compensation of effects between compressed zones and expanded zones (in the same or in different channels). For instance, an increase in the thickness of the diluate (which often provides the predominant resistance), in the whole channel or in a part of it, especially where the solution is less conductive, causes an increase in the average Ohmic resistance. Imbalances may also affect hydraulic friction, increasing the overall pressure drop. An increment in non-Ohmic resistance is another well-known detrimental effect of uneven flow rate distributions [13].

**Figure 1:**
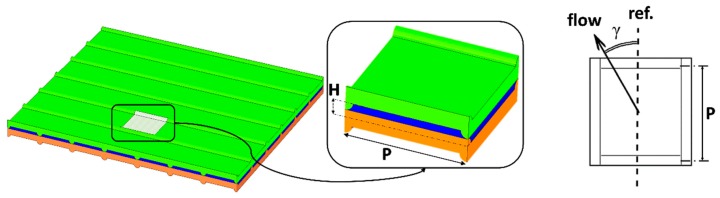
Profiled membranes of the Overlapped Crossed Filaments (OCF) type. The repetitive periodic unit of a cell pair is shown, enlarged, in the inset. The geometric parameters *H* (channel thickness), *P* (pitch) and *γ* (flow attack angle) are indicated.

All the aspects of practical interest examined in this section have provided the motivation to the present work. In particular, this paper goes inside the unexplored field of the TMP effects, taking a first step concerning mechanical response (deformation), flow and mass transfer characteristics at the local scale of a periodic unit. For this purpose, simulation tools implementing well-established and validated physical models and numerical methods were developed. Profiled membranes of the Overlapped Crossed Filaments (OCF) type were simulated. They are made by an array of semi-cylinders on both membrane sides, placed at 90° each other, as shown in Figure 1.

## 2. Results and Discussion

### 2.1. Mechanical Results

#### 2.1.1. Influence of Pitch to Height Ratio (*P*/*H*) and Limiting Values

**Figure 2:**
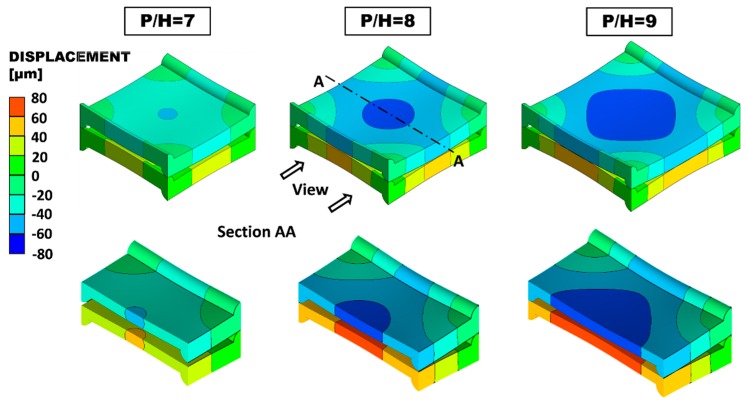
Deformation of membranes with different *P/H* ratios under *TMP* = +0.8 bar. The quantity shown is the displacement in the direction orthogonal to the undeformed membranes (*y*). Top: external view; bottom: view after sectioning by a mid-plane A-A.

Computational results for the deformation of cells with different pitch (*P*, distance between two profiles on the same membrane side) to channel height (*H*, distance between the two undeformed membranes) ratios under the conservative value of TMP = + 0.8 bar (the “+” sign refers to compression) are presented in Figure 2. Three values of *P*/*H* are considered (7, 8 and 9). The first contact between the two membranes approximately occurs for *P*/*H* = 9 and is located at the centres of the side ridges (note that an inter-membrane clearance of ~60 μm is still preserved at the centre of the periodic unit). Therefore, the value *P*/*H* = 8 was chosen as the largest admissible one.

#### 2.1.2. Membrane and Channel Deformation for the Selected Geometry (*P/H* = 8)

**Figure 3:**
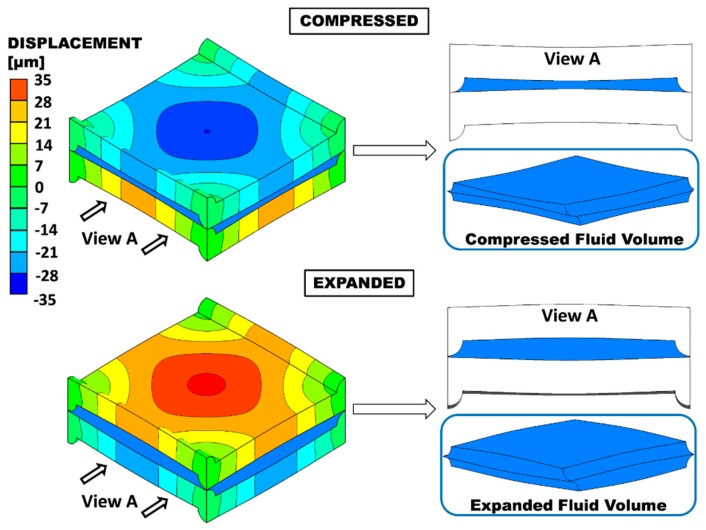
Deformation of membranes with *P/H* = 8 for the compressed and the expanded cases at TMP = ±0.4 bar. The quantity shown is the displacement in the direction orthogonal to the undeformed membranes (*y*). The corresponding deformed fluid volume is shown in the insets.

The geometry characterized by the maximum admissible *P*/*H* ratio (8) was subjected to *TMP* varying in 0.1 bar steps from −0.4 bar (expansion) to +0.4 bar (compression), and the corresponding deformation was computed. Figure 3 shows the deformed configuration for *P*/*H* = 8 under TMP = ±0.4 bar. The insets on the right show the deformed fluid volumes. The maximum relative variation of the clearance occurs at the centres of the ridges. Here, the distance between the two opposite membranes (thickness of the fluid passage), which is *H*/2 = 100 μm in the undeformed configuration, decreases to ~53.7 μm in the compression case and increases to ~148.4 μm in the expansion case. The distance between opposite membranes at the centre of the domain, which is *H* = 200 μm in the undeformed configuration, decreases to ~130 μm under compression or increases to ~272 μm in expansion (i.e., the maximum deflection at the centre is ~ ±70 μm).

**Figure 4:**
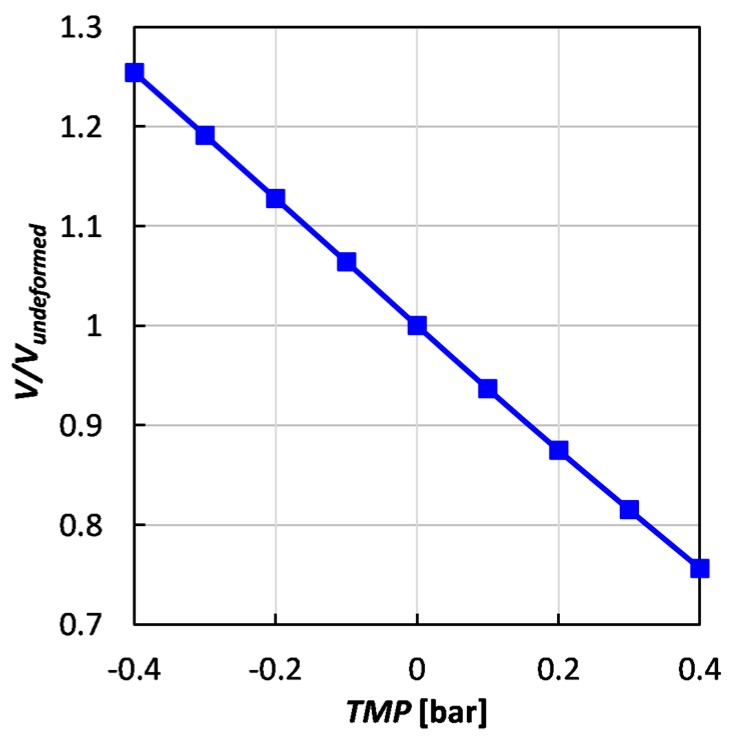
Fluid volume (normalized by the undeformed volume) as a function of trans-membrane pressure for *P/H* = 8.

Figure 4 provides information concerning the TMP effects, in the whole range studied, on the fluid volume. The volume follows a linear trend and exhibits an almost perfect symmetry between compression and expansion; the volume changes by ±25% for TMP = ±0.4 bar.

### 2.2. CFD Results for P/H = 8

#### 2.2.1. Undeformed Configuration

**Figure 5:**
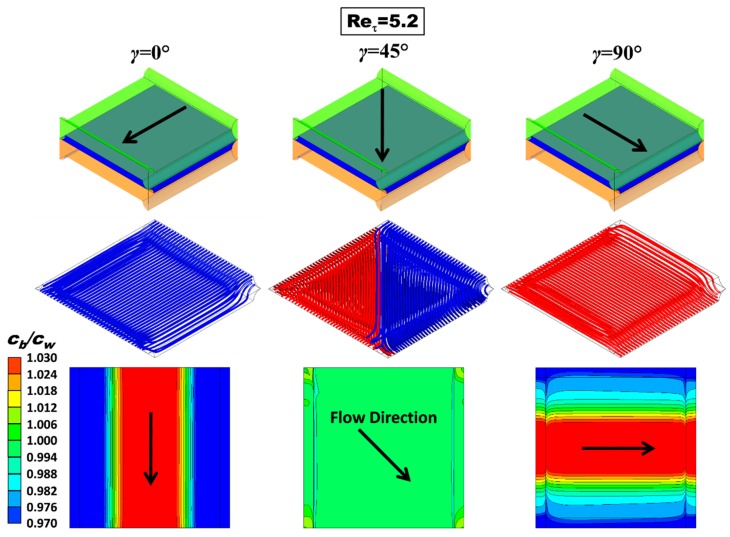
CFD results for the undeformed configuration with *P/H* = 8 at Re*_τ_* = 5.2 (approach velocity ~4 cm/s). Top row: sketches illustrating the flow direction; middle row: 3-D streamlines; bottom row: maps of the concentration polarization coefficient *θ = c_b_/c_w_* on the upper wall. *c_b_*= 500 mol/m^3^, flux corresponding to a current density of 50 A/m^2^ entering the fluid domain (dilute channel of RED or concentrate channel of ED).

Figure 5 shows 3-D streamlines and maps of the polarization coefficient *θ* = *c_b_*/*c_w_* (bulk to wall concentration ratio) in the undeformed configuration characterized by *P*/*H* = 8 for a friction velocity Reynolds number Re*_τ_* = 5.2 (bulk Reynolds number Re ≈ 17.6, approach velocity ~4 cm/s) and all three values of the flow attack angle (angle formed by the flow direction with the membrane ridges belonging to the upper wall) investigated (*γ* = 0°, 45° and 90°). The flow direction is indicated by arrows. Definitions of approach velocity and friction velocity Reynolds number are provided in Section 3.3.2, Equations (11) and (13).

The streamlines show that the flow is regular and parallel at this low value of Re. The corresponding plots for *γ* = 0° and *γ* = 90° are identical apart from a 90° rotation and a top-bottom reflection. For *γ* = 45°, streamlines were shown in two colours according to the face from which they enter the unit cell; the graph shows that there is no mixing between the two inlet streams. 

The maps of *θ* = *c_b_*/*c_w_* in the bottom row show that the case *γ* = 45° provides a more uniform distribution of the wall salt concentration, while the other two cases exhibit a very strong spanwise non-uniformity; the concentration is lower in the central region of the wall, where it becomes less than the bulk value despite the net overall salt flux being into the channel, and larger in the lateral regions of the channel walls, where low fluid velocities (stagnation zones) occur. Please note that the distribution of *θ* on the upper wall for *γ* = 0°, once rotated by 90°, would become the corresponding lower wall distribution for *γ* = 90° and *vice versa*. Also, remember that the values of the polarization coefficient depend on the flux imposed at the boundary and on the bulk concentration considered. Therefore, for example, much lower values would be obtained for dilute solutions.

#### 2.2.2. Deformed Configurations

**Figure 6:**
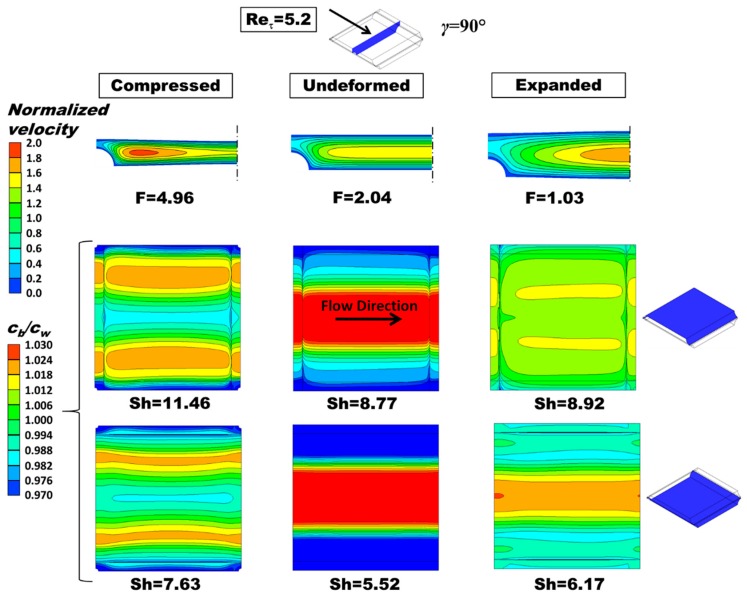
Influence of deformation on flow and mass transfer for *P/H* = 8, *γ* = 90°. Left column: compressed (TMP = +0.4 bar); middle column: undeformed; right column: expanded (TMP = −0.4 bar). Top row: distribution of the streamwise velocity component in the central cross section of the channel (for symmetry reasons, only half map is shown); middle and bottom rows: distribution of the polarization coefficient on the upper and lower walls (see sketches on the right). *c_b_* = 500 mol/m^3^, flux corresponding to a current density of 50 A/m^2^ entering the fluid domain (dilute channel of RED or a concentrate channel of ED). *F* ratio and Sherwood number are also reported.

For the sake of brevity, the influence of deformation on flow and mass transfer in OCF membranes with *P*/*H* = 8 is illustrated here in Figure 6 only for a friction velocity Reynolds number Re*_τ_* = 5.2 (corresponding to bulk Reynolds numbers between ~7 and ~35, approach velocity ~1.6 and ~7.8 cm/s, depending on the load conditions) and a flow attack angle *γ* = 90° (flow orthogonal to the profile ridges adjacent to the upper wall of the fluid channel), as evidenced in the inset.

Three configurations are examined: compressed by a trans-membrane pressure TMP = +0.4 bar (left column), undeformed (middle column), and expanded by a trans-membrane pressure TMP = −0.4 bar (right column). The top row reports contour plots of the velocity component along the main flow direction in the central cross section of the channel, while the middle and bottom rows report contour plots of the polarization coefficient *θ* = *c_b_*/*c_w_* on both the upper and the lower wall of the fluid-filled channel, as clarified by the sketches in the rightmost part of the figure. The corresponding values of the *F* ratio (friction coefficient normalized by that for laminar flow in an undeformed void plane channel of indefinite width, 96/Re) and of the Sherwood number are also reported.

In the deformed channels, the normalized axial velocity component exhibits larger maximum values, which are located closer to the longitudinal ridges in the case of the compressed channel.

The *F* ratio increases from ~2.04 to ~4.96 with compression and decreases from ~2.04 to ~1.03 with expansion. In regard to the Sherwood numbers that on the upper wall (flow orthogonal to the profile ridges) increases significantly with compression (from 8.77 to 11.46, i.e., by ~30%) and increases, but negligibly, also with expansion (from 8.77 to 8.92, i.e., by ~2%). That on the lower wall (flow parallel to the profile ridges) increases significantly with compression (from 5.52 to 7.63, i.e., by ~38%) and increases less, but still appreciably, also with expansion (from 5.52 to 6.17, i.e., by ~12%). 

For greater readability of the results, the dimensioned values of the approach velocity *U* and of the mass transfer coefficient *k* for the three conditions in Figure 6 (TMP = 0 or ±0.4 bar) are summarized in Table 1. In all three cases the friction velocity Reynolds number Re*_τ_* is ~5.2, corresponding to an inlet-outlet pressure drop in a unit cell (1.6 mm in side) of ~34.36 Pa.

Distributions of the polarization coefficient *θ* = *c_b_*/*c_w_* are deeply affected by deformation. In the compressed configuration, both on the upper and on the lower wall the region of high *θ* (i.e., low concentration) observed in the undeformed case splits into two smaller regions, symmetrically located about the midline parallel to the flow direction, whereas the central region of the wall close to this midline exhibits low values of *θ* (i.e., high values of concentration). In the expanded configuration, the concentration distribution on the lower wall remains similar to that observed in the undeformed case, with a single large central strip where *c_w_* < *c_b_*, which is consistent with the fact that the longitudinal velocity exhibits a single central maximum as in the undeformed case (see top row). The *θ* distribution on the upper wall becomes flat, with two shallow *θ* maxima (i.e., *c_w_* minima) symmetrically located about the longitudinal midline.

By comparing the polarization coefficient maps and the velocity maps in Figure 6, it can be observed that under the present assumption of mass flux entering the channel, higher concentration levels on the wall correspond to stagnation regions, whereas low values of concentration occur in regions of high streamwise velocity as an effect of axial advection.

#### 2.2.3. Global Parameters

Among the performance parameters of greatest interest which can be affected by deformation, we selected the friction coefficient and the Sherwood number [16,50].

**Figure 7:**
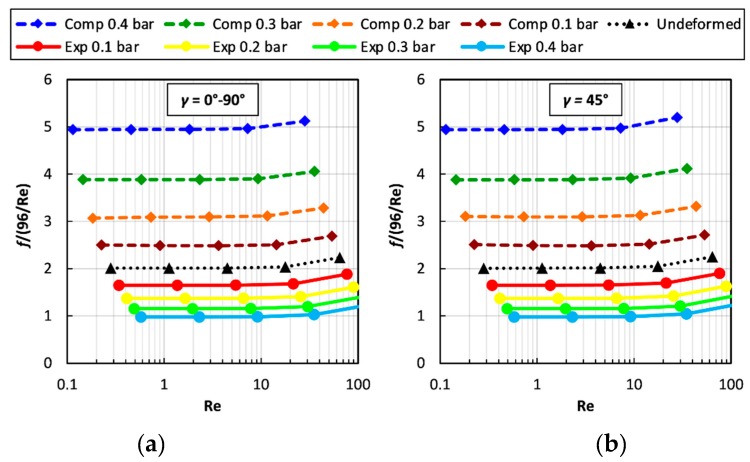
Normalized Darcy friction coefficient (*F* ratio) as a function of Re for *P/H* = 8, different values of the trans-membrane pressure TMP and two values of the flow attack angle *γ*. (**a**) *γ* = 0° or 90°; (**b**) *γ* = 45°.

Figure 7 reports the normalized Darcy friction coefficient, i.e., the *F* ratio, as a function of Re for *P*/*H* = 8 at different values of *TMP*. Graph (a) is for flow attack angles *γ* of 0° or 90° (equivalent in regard to friction), while graph (b) is for *γ* = 45°. Please note that the results of each series of simulations performed at a given Re*_τ_* appear as an inclined row of symbols since they correspond to different values of Re.

For any *γ* and applied TMP, *F* is flat up to Re ≈ 10, indicating that inertial effects are negligible (self-similar flow). A significant departure from the void channel behaviour is observed only for Re»10. The influence of TMP is to enhance friction under compression and to reduce it under expansion. This effect is expected because, for any given Re, in a compressed channel the cross section is reduced, local velocities increase and thus pressure drops are higher (the opposite occurs in an expanded channel). For the same absolute value of TMP, the influence of compression is slightly larger than that of expansion: TMP = +0.4 bar leads to an increase in *F* by a factor of ~2.5, while TMP = −0.4 bar leads only to a halving of *F*. The influence of the angle *γ* is negligible (graphs (a) and (b) are practically identical), indicating a substantial isotropy of the profiled membrane lattice in terms of hydraulic friction. This behaviour is typical in the case of low Reynolds numbers, as largely documented in the literature [10,16,50,51].

**Figure 8:**
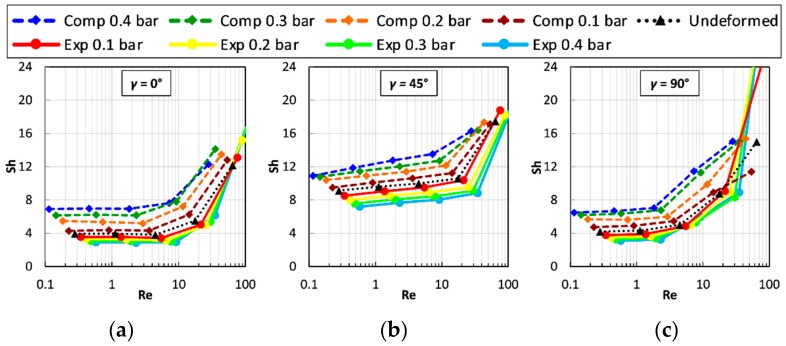
Sherwood number on the upper wall as a function of the Reynolds number for *P/H* = 8 and different values of the trans-membrane pressure and of the flow attack angles. (**a**) *γ* = 0°; (**b**) *γ* = 45°; (**c**) *γ* = 90°.

Figure 8 reports the Sherwood number on the upper channel wall, for *P*/*H* = 8 as a function of the Reynolds number and for different values of the trans-membrane pressure. Graphs (a), (b) and (c) are for flow attack angles *γ* of 0°, 45° and 90°, respectively. Please note that the cases *γ* = 0° and 90° are equivalent in regard to friction but not in regard to mass transfer on a specified wall. However, for symmetry reasons, the Sherwood number on the lower wall of the channel at a given *γ* is identical to that on the upper wall at the complementary flow attack angle 90° − *γ* (also the distributions of wall quantities such as concentration and mass transfer coefficient would be the same, apart from rotations and reflections). Therefore, values of Sh for the lower wall were not separately reported.

When *γ* = 0°, Figure 8(a), for any applied *TMP* the Sherwood number on the upper wall changes little with Re up to ~10, while for *γ* = 90°, Figure 8(c), the departure from this flat behaviour occurs earlier (Re ≈ 2). For *γ* = 0° or 90°, the Sherwood number at low Reynolds numbers ranges between ~3 and ~7 and thus is less than the theoretical value for a void plane channel of indefinite width (~8.24 under uniform mass flux conditions [52]). This indicates that in this Reynolds number range, the “shadow” effects of the profiles hinder mass transfer. The behaviour of Sh is different for a flow attack angle of 45°, Figure 8b, for which, even at very low Reynolds numbers, Sh increases with Re and is larger than in a void channel for all compressed configurations, while it becomes slightly lower only for the expanded ones. Under all conditions, Sh increases rapidly as Re exceeds some critical value and, at Re ≈ 30–100, it becomes much larger than in a void channel. The most peculiar behaviour is exhibited by the upper wall Sherwood number in the expanded cases and *γ* = 90°, which jumps to very high values (up to ~40 for TMP = −0.4 bar) as Re exceeds ~50 due to the increasing importance of flow recirculation.

The influence of trans-membrane pressure on Sh is more complex than that on *F*. On the whole, compression enhances mass transfer and expansion reduces it; the influence of channel deformation on mass transfer is less marked than on friction. Some anomalous behaviour of Sh is observed only in the cases characterized by *γ* = 90° and Re > ~50, in which the highest values of Sh are obtained for the largest expansion. Under all deformation conditions, the flow orientation *γ* = 45° yields the highest values of Sh. This is in contrast with the behaviour of the friction coefficient, see Figure 7, which is only minimally affected by the flow attack angle.

## 3. Materials and Methods

### 3.1. Simulation Strategy

In the present study, membrane deformation was computed by the Finite Element (FE) Ansys-Mechanical software (ANSYS, Inc., Canonsburg, PA, USA, version 18.1), while fluid dynamics and mass transfer in the deformed channels were computationally investigated by the Finite Volume (FV) Ansys-CFX software (ANSYS, Inc., Canonsburg, PA, USA, version 18.1). To reduce the computational effort, only one fluid channel at a time was simulated and the local pressure difference between adjacent channels was applied as a boundary condition. Simulations were conducted for a periodic unit as shown in Figure 1, exploiting the periodic nature of the profiled membrane geometry by the approach discussed in detail in previous papers [51] and summarized in Section 3.3.1. The analysis was carried out in three steps:

1. First, the influence of the pitch-to-height ratio (*P*/*H*) was addressed by mechanical simulations. A TMP of 0.8 bar was applied, and the geometry with the largest value of *P*/*H* still able to withstand this load without collapsing (i.e., without exhibiting a contact between opposite membranes) was identified. The figure of 0.8 bar was conservatively chosen as a value comfortably larger than the highest TMP actually expected in real RED/ED applications. The search for the largest admissible *P*/*H* was motivated by the fact that small values of *P*/*H* are associated with large pressure drops: many studies [13,16,30,53,54,55] have highlighted the importance of reducing pressure drop and thus mechanical power losses in the channels, especially in RED applications. It is true that the increase of *P*/*H* may also cause a reduction in mass transfer coefficients, but its effect on stack performance is usually less important.
2. The geometry thus identified was then investigated under expansion and compression conditions corresponding to TMP varying from −0.4 to +0.4 bar. As discussed in the Introduction, this range encompasses most of the conditions that are likely to occur in actual ED/RED applications. For each load condition, the deformed configuration was computed by mechanical simulations.
3. Finally, for each deformed configuration, fluid flow and mass transfer in the expanded or compressed channel were numerically simulated by CFD; in particular, friction coefficients and Sherwood numbers were computed as functions of the Reynolds number.

**Figure 9:**
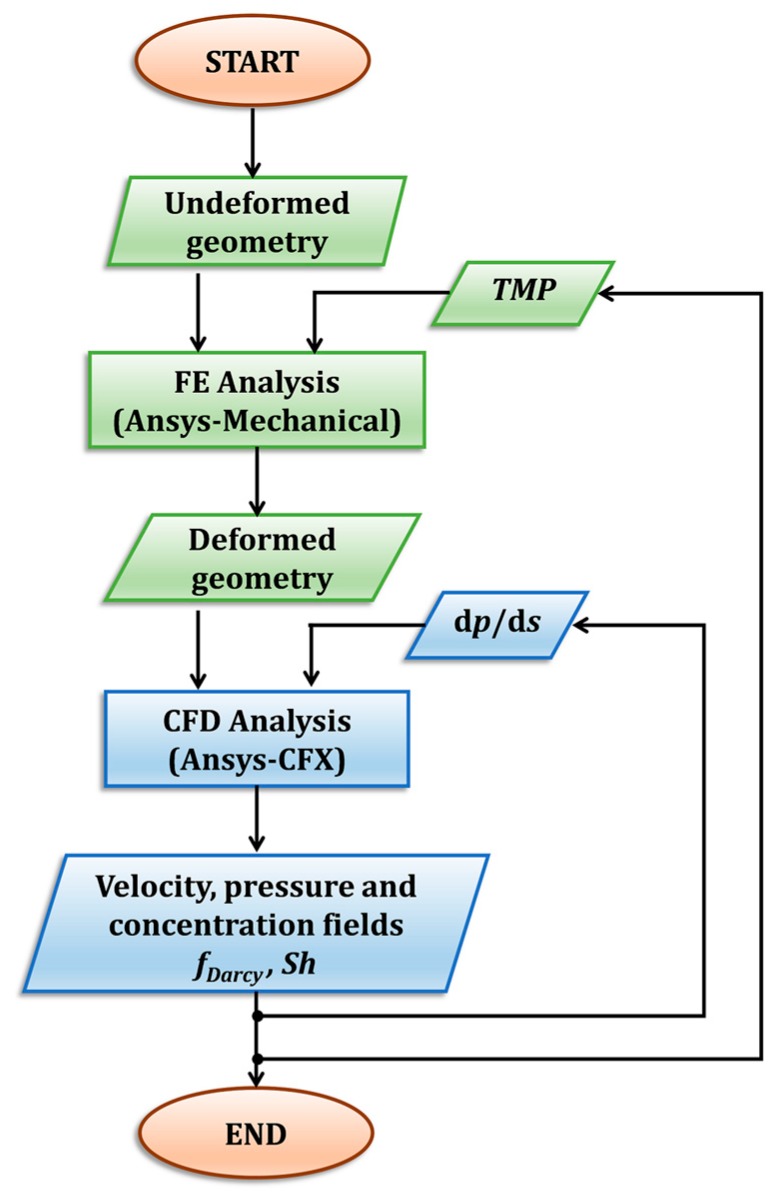
Flow chart of the computational process for any chosen geometry.

Steps 2–3 can be schematically represented by the flow chart in Figure 9. It is worth noting that the present modelling approach can also be applied to the simulation of membrane/spacer systems, provided suitable changes and adjustments are made in some specific aspects (e.g., concerning the boundary conditions).

Note also that in the present study, the coupling between fluid and structure is only one-way (open-loop model): a given TMP causes channel deformation, which, in its turn, causes changes in the flow field and thus in pressure losses and mass transfer coefficients. Changes in TMP induced by the flow field, which would close the fluid-structure interaction loop, are not considered at this stage: they cannot be predicted at periodic unit level but only by considering a larger scale (stack level), where they are mainly caused by inlet-to-outlet pressure gradients.

### 3.2. The Mechanical Problem

The mechanical properties of a membrane depend on manufacturing method, nature of co-polymers, cross-linking degree, ageing, etc. In the present study, cation and anion exchange membranes were assumed to exhibit the same mechanical properties and, for the sake of simplicity, were treated as linearly elastic, homogeneous and isotropic media. Representative values of 150 MPa for the Young modulus (*E*) and 0.4 for the Poisson ratio (*ν*) were chosen for the membranes among literature data broadly ranging from 10 MPa to 1 GPa for *E* (see the Introduction) and from 0.25 to 0.4 for *ν* [39,41]. 

The linearly elastic hypothesis is quite reasonable for ED/RED membranes within the mild load conditions considered in this study: for TMP = ±0.4 bar, the maximum computed von Mises stress is ~2 MPa, which is below the limit stress for linearly elastic behaviour generally exhibited by ion exchange membranes [39,40,41,42,43], including those tested in our experiments (see Figure S1 in the Supplementary Materials). 

The homogeneity assumption is based on the membrane structure and on the preparation technique adopted, as described by the manufacturer (FujiFilm Europe, Tilburg, The Netherlands).

In regard to the isotropy assumption, tests conducted by the authors evidenced a maximum difference of ~15% between the values of the Young modulus along the MD (machine direction) and CD (cross direction) axes, both for cation and anion exchange membranes. In view of this modest degree of anisotropy, we felt that taking this feature into account would have unnecessarily complicated the computations and multiplied the number of representative test cases without changing the results to any significant extent.

#### 3.2.1. Governing Equations

Equilibrium, compatibility and constitutive equations were numerically solved in order to find the deformed configuration of the body [56]. They are quite standard but, for the sake of completeness, are reported here below in Cartesian tensor notation (no summation over repeated indexes).

Equilibrium:

(1) $$\frac{\partial \sigma_{i}}{\partial x_{i}}+\frac{\partial \tau_{ij}}{\partial x_{j}}+\frac{\partial \tau_{ik}}{\partial x_{k}}+F_{i}=0$$

Compatibility:

(2) $$\frac{\partial^{2}\varepsilon_{i}}{\partial x_{j}^{2}}+\frac{\partial^{2}\varepsilon_{j}}{\partial x_{i}^{2}}=\frac{\partial^{2}\gamma_{ij}}{\partial x_{i}\partial x_{j}};\;2\frac{\partial^{2}\varepsilon_{k}}{\partial x_{i}\partial x_{j}}=\frac{\partial }{\partial x_{k}}\left(\frac{\partial \gamma_{jk}}{\partial x_{i}}+\frac{\partial \gamma_{ik}}{\partial x_{j}}-\frac{\partial \gamma_{ij}}{\partial x_{k}}\right)$$

Constitutive:

(3) $$\varepsilon_{i}=\frac{1}{E}\left[\sigma_{i}-\nu \left(\sigma_{j}+\sigma_{k}\right)\right];\;\gamma_{ij}=\frac{1}{G}\tau_{ij}$$

where $\sigma_{i}$ are normal stresses, $\tau_{ij}$ are shear stresses, *F_i_* are body forces, $\varepsilon_{i}$ are normal strains, $\gamma_{ij}$ are shear strains, and *G* = *E*/[2 × (1 + *ν*)] (shear modulus). The small deformation approximation was not used. 

#### 3.2.2. Computational Domain and Boundary Conditions

The computational domain for the mechanical simulations is the periodic unit shown in the central inset of Figure 1. Please note that the planform of this unit is a square. The undeformed channel thickness *H* was assumed to be 0.2 mm and the undeformed membrane thickness 0.12 mm; these values are representative of advanced membrane-channel configurations currently being considered for ED and RED applications [1,46,47,57]. Profiles of adjacent membranes were assumed to be aligned on top of one another; in practice, this arrangement may not be precisely achieved since, in operation, shifts would be likely to occur.

**Figure 10:**
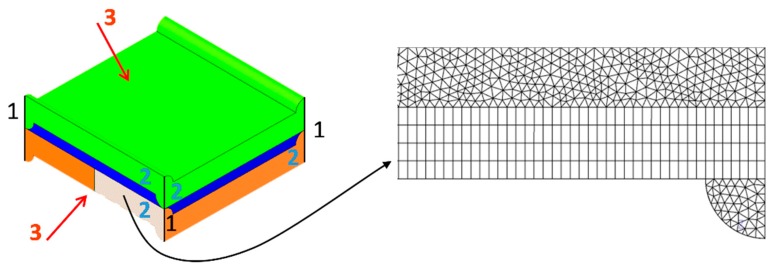
Computational domain. Numbers 1–3 indicate the mechanical boundary conditions (see text). A detail of the finite element mesh is shown on the right.

For the sake of clarity, geometrical and mechanical quantities are summarized in Table 2. The computational domain is also shown, enlarged, in Figure 10, where the mechanical boundary conditions are evidenced:

1. Each of the four segments representing the external vertical edges of the domain (1) was clamped, i.e., zero displacement and rotation were imposed to all points belonging to it.
2. Each of the four side faces of the domain (2) was imposed zero displacement in the direction normal to itself, so that a single computational domain is representative of a periodic array of repetitive units.
3. The trans-membrane pressure TMP (relative to that of the internal fluid channel) was applied to the whole outer surface of the domain (3). Please note that TMP > 0 for compression conditions, while TMP < 0 for expansion conditions.

#### 3.2.3. FE mesh for Mechanical Simulations

A detail of the finite element mesh is shown in Figure 10. The use of a hybrid (hexahedral-tetrahedral) grid was necessary. Grid dependence was preliminary assessed; Table 3 reports the maximum displacement at the outer surface of the domain computed for TMP = 0.8 bar and *P*/*H* = 8 with increasingly fine meshes.

On the basis of these results, computational meshes of 500 × 10^3^ elements (OCF-II) were used in all following simulations as a compromise between accuracy and computational effort.

#### 3.2.4. Mechanical Model Validation

**Figure 11:**
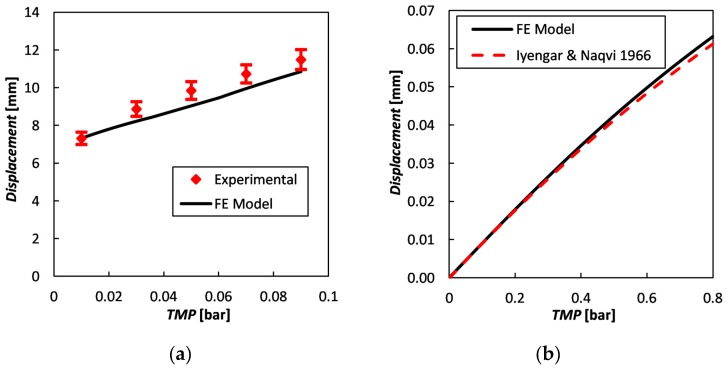
Maximum displacement for a square, edge-clamped membrane as a function of the trans-membrane pressure. Comparison of FE predictions (solid line) with (**a**) experimental results (symbols) of bulge tests on a 10 × 10 cm^2^ sample and (**b**) the first-order analytical solution by Iyengar and Naqvi [58] for a 2 × 2 mm^2^ membrane (dashed line).

The FE model was first validated by comparison with existing experimental bulge test results, obtained in the context of an independent study for 10 × 10 cm^2^ square samples of flat ion exchange membranes. Details of the experiments are reported in Section 3 of the Supplementary Material (Figure S2). Figure 11a compares the predicted and experimental maximum displacements (placed at the central point of the membrane) as functions of the trans-membrane pressure. Error bars are reported for the experimental data. A good agreement can be observed, with a maximum relative discrepancy of a few percent.

A further validation of the FE model was performed by comparing numerical predictions with an analytical solution of structural mechanics for a two-dimensional domain [58]. A square body loaded with a uniform pressure and with all the edges clamped was considered. Since the membrane deflection overcomes the “small deflection” range, a suitable analytical solution was used for the comparison. In particular, Figure 11b reports the maximum deflection as a function of the trans-membrane pressure. The broken line is the first-order approximated analytical solution reported by Iyengar and Naqvi [58], the solid line is the present FE numerical solution. In this case, the square membrane is 2 mm wide and 0.120 mm thick and Young’s modulus is 150 MPa. The Poisson ratio is 0.316, as the analytical solution proposed in reference [58] was specifically obtained for this value. Figure 11b shows that our numerical simulations are in good agreement with the approximate theoretical solution, the discrepancy increasing with TMP and being only ~3% at 0.8 bar.

### 3.3. The Fluid Dynamics and Mass Transfer Problem

#### 3.3.1. Computational Approach 

The fluid channel was simulated by CFD in the undeformed, compressed and expanded configurations in order to evaluate its pressure drop and mass transport performance. The ionic transport was simulated assuming the local electroneutrality condition in the whole fluid domain. Under this hypothesis, from the Nernst-Planck equations and the mass balances of the two ions of a binary electrolyte, a convective-diffusive transport equation can be derived [51,59,60,61]. This simplifies the calculations, requiring only the need for a choice concerning the boundary condition at the membrane-solution interface (uniform concentration, uniform flux, or mixed condition); however, the influence of the boundary conditions on the mass transfer coefficient is small [8,52]. Please note that the potential is eliminated from the transport equation, and therefore the electric field and associated phenomena (e.g., Ohmic resistance) are not calculated by this simulation approach. Moreover, electroneutrality conditions are assumed and the electric double layer at the membrane-solution interface is not simulated, so that special conditions in which an extended space charge region occurs (e.g., electroconvection under overlimiting conditions) are not taken into consideration. 

The governing equations were solved by the finite volume code Ansys-CFX^®^. From the numerical solution of these equations, velocity, pressure and electrolyte concentration fields are obtained. Raw results are then elaborated in order to calculate friction factor and Sherwood number. This simulation method is particularly suitable for the implementation of integrated multi-scale process simulators [1], where basic data produced by CFD are merged with higher-scale simulation tools [8,16,62].

#### 3.3.2. Governing Equations and Definitions

For the fluid dynamics/mass transfer problem the assumptions are those of steady laminar flow and constant-property fluid. The former assumption is amply justified by the low Reynolds numbers (<100 in most cases), the latter by the small concentration changes occurring in a generic unit cell (polarization coefficients *c_b_*/*c_w_* ranging from 0.97 to 1.03, see Figure 5 and Figure 6). Under these assumptions, we used the most complete possible model, i.e., that made up the full, three-dimensional continuity, Navier-Stokes and scalar transport equations. 

The continuity equation (with implicit summation) is simply

(4) $$\frac{\partial u_{i}}{\partial x_{i}}=0$$

where *u_i_* is the *i*-th velocity component of the fluid.

As anticipated in Section 3.1., all simulations were carried out under the hypothesis of fully developed flow and concentration field, thus simulating the Unit Cell [51]. In this approach, periodic boundary conditions are imposed to all variables between the inlet and outlet faces of the computational domain. At the same time, it is necessary to allow for the variation of pressure and bulk concentration along the main flow direction ***s***, due to frictional losses and solute inflow or outflow through the channel walls, respectively. These apparently contradictory requirements are reconciled as follows.

- Consider pressure *p* first. In the fully developed region of a channel, *p* can be decomposed into a periodic component $\tilde{p}$, whose spatial distribution repeats itself identically in each unit cell, and a large-scale component −*K_p_*(**x**·**s**) which decreases linearly along the main flow direction whose unit vector is **s** (**x** is the position vector of components *x_i_*). By substituting $\tilde{p}-K_{p}\left(x\cdot s\right)=\tilde{p}-K_{p}x_{i}s_{i}$ for *p* in the *i*-th steady-state Navier-Stokes equation:
  (5) $$\frac{\partial \rho u_{j}u_{i}}{\partial x_{j}}=-\frac{\partial p}{\partial x_{i}}+\frac{\partial }{\partial x_{j}}\mu \frac{\partial u_{i}}{\partial x_{j}}$$
  (where *ρ* and *μ* are the fluid’s density and viscosity), it becomes
  (6) $$\frac{\partial \rho u_{j}u_{i}}{\partial x_{j}}=-\frac{\partial \tilde{p}}{\partial x_{i}}+\frac{\partial }{\partial x_{j}}\mu \frac{\partial u_{i}}{\partial x_{j}}+K_{p}s_{i}$$
  Equation (6) is similar to Equation (5), but (a) the “true” pressure *p* is replaced by its periodic component $\tilde{p}$, and (b) a body force per unit volume (mean pressure gradient) acting along the main flow direction **s** appears at the right hand side. If required, the “true” pressure *p* can always be reconstructed from the simulation results as *p* = $\tilde{p}-K_{p}\left(x\cdot s\right)$.
- In regard to the concentration *c*, by definition of fully developed conditions it can be decomposed into a periodic component $\tilde{c}$ and a large-scale component *K_c_*(**x**·**s**), where *K_c_* can now be either positive (net inflow of electrolyte into the channel) or negative (net outflow of electrolyte from the channel). By substituting $\tilde{c}+K_{c}\left(x\cdot s\right)=\tilde{c}+K_{c}x_{i}s_{i}$ for *c* in the transport equation
  (7) $$\frac{\partial u_{j}c}{\partial x_{j}}=\frac{\partial }{\partial x_{j}}D\frac{\partial c}{\partial x_{j}}$$
  (where *D* is the electrolyte diffusivity), after some manipulation one obtains:
  (8) $$\frac{\partial u_{j}\tilde{c}}{\partial x_{j}}=\frac{\partial }{\partial x_{j}}D\frac{\partial \tilde{c}}{\partial x_{j}}-K_{c}u_{s}$$
  in which *u_s_* = **u**·**s** is the local velocity component along the main flow direction ***s***. The large-scale gradient *K_c_* can be obtained by an elementary balance as:
  (9) $$K_{c}=\frac{A}{V}\frac{j}{u_{s}}$$
  in which *j* is the molar salt flux at walls (imposed in the simulation), *A* is the membrane surface active area in a fluid unit cell (so that *jA* is the molar flow per unit time, mol/s), *V* is the cell volume and 〈*u_s_*〉 is the volume average of *u_s_*.

In the present study, the (bulk) Reynolds number was conventionally defined as:

(10) Re=ρU2Hμ

i.e., it was based on the hydraulic diameter 2*H* of a void (profile-less) and undeformed channel of thickness *H* in the limit of indefinite width, and on the approach velocity

(11) $$U=\frac{Q}{S}$$

in which *Q* is the volume flow rate through a cross section of the channel orthogonal to the main flow direction and *S* is the cross sectional area of a void (profile-less) and undeformed channel of thickness *H*. The above definitions of *U* and Re are consistent with those adopted in our previous works on undeformed spacer-filled channels or profiled membranes [10,16,50,51].

The Darcy friction coefficient *f_Darcy_* was defined with reference to the above approach velocity *U* and hydraulic diameter 2*H*, i.e., as:

(12) $$f_{Darcy}=\left|\frac{dp}{ds}\right|\frac{4H}{\rho U^{2}}$$

In the simulations, the driving pressure gradient *K_p_* = |d*p*/d*s*| in Equation (6) was imposed, while the flow rate was obtained as part of the solution. Please note that |d*p*/d*s*| can be expressed in terms of the friction velocity Reynolds number

(13) Reτ=uτρμH2

in which *u_τ_* is the friction velocity,

(14) $$u_{\tau }=\sqrt{\frac{H}{2\rho }\left|\frac{dp}{ds}\right|}$$

Therefore, in the parametrical analyses illustrated above, results were obtained for a given Re*_τ_* (friction velocity Reynolds number) rather than for a given Re (bulk Reynolds number). Please note that according to the present definitions, between Re, Re*_τ_* and *f_Darcy_* the following relation holds:

(15) fDarcy=128(ReτRe)2

To separate the effects of profile shape and channel deformation from the effects of varying the flow rate (and thus Re), the Darcy friction coefficient was normalized by that holding for parallel laminar flow in a void plane channel of indefinite width, i.e., 96/Re. Therefore, the following quantity (*F*-ratio) was reported:

(16) F=fDarcy96/Re

The local concentration polarization coefficient *θ* was defined as:

(17) $$\theta =\frac{c_{b}}{c_{w}}$$

where *c_b_* is the molar bulk concentration and *c_w_* is the local molar concentration at the membrane surface. Please note that defining the average polarization coefficient in such a way that it is lower than 1 [10,16,51], the local polarization coefficient in Equation (17) refers to the case of either a dilute channel of RED or a concentrate channel of ED, where the flux enters from membrane’s walls.

The Sherwood number was defined as

(18) $$Sh=\frac{jA}{\left(c_{b}-\langle c_{w}\rangle \right)A_{proj}}\frac{2H}{D}$$

in which *A_proj_* is the projected membrane surface area and (*c_w_*) is the area average of *c_w_* on the same membrane. Please note that the Sherwood numbers on the two membranes facing a channel may differ depending on the flow direction.

#### 3.3.3. Flow Attack Angle, Boundary Conditions and Simulation Settings

The flow attack angle *γ* is defined (Figure 1) as the angle formed by the flow direction with the membrane ridges belonging to the upper wall of the channel under consideration. In this study, the two values *γ* = 0° and 45° were considered; for symmetry reasons, the case *γ* = 90° exhibits the same friction coefficient as the case *γ* = 0°, while the surface distributions of local quantities such as concentration, mass transfer coefficient (Sherwood number) and polarization coefficient on the upper and lower walls of the channel simply exchange place and rotate by 90° with respect to the case *γ* = 0°. 

As mentioned in discussing the unit cell approach, translational periodicity was imposed for **u**, *p* and *c* between opposite inlet-outlet boundaries. At the membrane surfaces, no slip conditions were imposed for velocity and a uniform value of 2.6 × 10^−4^ mol/(m^2^s) for the molar salt flux entering the fluid, corresponding to a current density of 50 A/m^2^. An NaCl aqueous solution at a bulk concentration of 500 mol/m^3^ was considered (i.e., seawater, see physical properties reported in Table 4). Please note that these choices on flux and bulk concentration affect directly the polarization coefficient (Equation (17)), while, the Sherwood number depends only on geometry, Re and Sc, due to the linearity of the transport Equation (8).

#### 3.3.4. FV Mesh for CFD Simulations

Mainly hexahedral meshes were adopted in the CFD simulations. Small regions at the corners of the domain were discretized by tetrahedra (~4.4% of the total volumes), pyramids (~0.13%) and wedges (~0.03%). Grid dependence was evaluated for *P*/*H* = 8 in the undeformed configuration at Re*_τ_*≈ 5, corresponding to a bulk Reynolds number of ~20. Therefore, the test case selected for the grid-independence assessment lies well above the creeping flow range and close to the highest Reynolds numbers investigated. Results are shown in Table 5, where the computed values of the Darcy friction coefficient *f_Darcy_* and of the Sherwood number Sh are reported as functions of the number of finite volumes.

**Figure 12:**
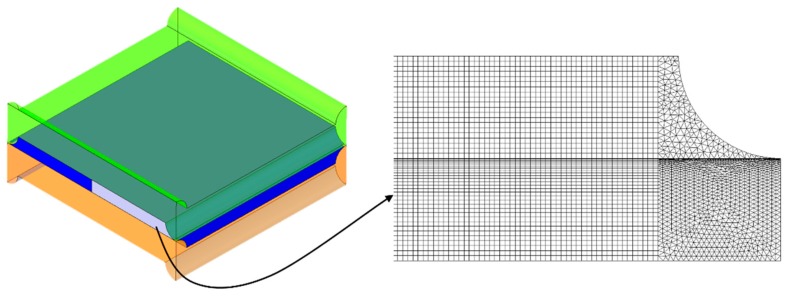
Details of the mesh chosen for the final simulations (undeformed domain, *P*/*H* = 8).

The mesh adopted for the final simulations (OCF-B) was characterized by about 4 million volumes. The channel height *H* was resolved by 40 finite volumes. Details of the same mesh are presented in Figure 12.

All simulations were conducted in double precision. The “High Resolution” (higher-order upwind) interpolation scheme was adopted for the advection terms. In regard to convergence, iterations were interrupted when the residuals of all variables became less than 10^−10^.

## 4. Conclusions

Integrated mechanical and fluid dynamics simulations were performed for profiled membranes of the OCF (“overlapped crossed filaments”) type. The membranes were treated as linearly elastic, homogeneous and isotropic, and values of the Young modulus and of the Poisson ratio representative of ion exchange membranes’ features (*E* = 150 MPa, *ν* = 0.4) were adopted.

Under these assumptions, the largest value of the pitch to height ratio withstanding a trans-membrane pressure of 0.8 bar without collapsing (i.e., without exhibiting a contact between opposite membranes) was found to be *P*/*H* = 8.

The influence of *TMP* (which was investigated here in the range −0.4–+0.4 bar) is an increase of friction under compression conditions and a reduction of it (although to a lesser extent) under expansion conditions. This imbalance of effects may produce an increase of total pressure drop in the stack. The influence of the flow attack angle is negligible, indicating a substantial hydrodynamic isotropy of the profiled membrane lattice at low Reynolds numbers. 

The influence of TMP on the Sherwood number is more complex. On the whole, compression enhances mass transfer and expansion reduces it; the influence of channel deformation on mass transfer is less marked than on friction. Some anomalous behaviour of Sh is observed in the cases characterized by *γ* = 90° and Re > ~50, in which the highest Sh is obtained for the largest expansion.

This study shows that *TMP* values of practical interest for ED/RED units can produce significant effects on deformation, flow and concentration fields (and, thus, on hydraulic friction and mass transfer coefficient). In general, other important quantities, e.g., Ohmic resistance, non-Ohmic resistance and limiting current density, will also be affected.

As far as membrane manufacturers are concerned, the main suggestion arising from this study is probably that stiffer membranes should be preferred in order to reduce the undesirable effects of membrane deformation, which result in an impairment of the ED/RED equipment performance. On the other hand, the current trend is towards the reduction of membrane thickness, mainly sought in order to reduce Ohmic losses. Therefore, producing membranes with the highest possible Young modulus (provided the membrane’s electrochemical behaviour is not impaired) will probably become a priority.

At a larger scale, combinations of geometric/mechanical features and operating conditions expose ED/RED stacks to the risk of severe local deformations, providing a new set-up, different from the nominal undeformed one, where all parameters are distributed as a result of a fluid-membrane mechanical interaction. Therefore, the actual process performance may be heavily affected by an imbalance of effects between compressed and expanded zones. This implies that the design and the performance prediction of devices under realistic operating conditions should take into account the membranes’ mechanical features.

From this perspective, correlations describing (a) the dependence of deformation on trans-membrane pressure and (b) the dependence of friction coefficients and Sherwood numbers on deformation, derived from the results of the present modelling approach, will be implemented into higher-scale (stack-level) models in order to close the fluid-structure interaction loop and characterize the amount and effects of maldistribution phenomena. Moreover, the simulation method presented in this study can be applied to the more traditional configurations of flat membranes with net spacers by suitable adjustments.

## Figures and Tables

**Table 1:** Approach velocity and mass transfer coefficients for the load conditions in Figure 6.

Quantity	Compressed +0.4 Bar	Undeformed	Expanded −0.4 Bar
[cm/s]	~1.6	~4	~7.8
〈*k*〉, upper wall [m/s]	~3.72 × 10^−5^	~2.84 × 10^−5^	~2.89 × 10^−5^
〈*k*〉, lower wall [m/s]	~2.47 × 10^−5^	~1.78 × 10^−5^	~2.00 × 10^−5^

**Table 2:** Geometrical and mechanical quantities.

Quantity	Value	Units
Membrane Young modulus, *E*	150	MPa
Membrane Poisson ratio, *ν*	0.4	-
Membrane thickness	120	μm
Channel thickness, *H*	200	μm
Pitch-to-height ratio, *P/H*	7–9	-
Angle between filaments	90	deg

**Table 3:** Grid dependence results (TMP = +0.8 bar).

FE Mechanical Mesh	No. Elements (*P*/*H* = 8)	Maximum Displacement at Membrane Surface [μm]
OCF-I	200 × 10^3^	67.04
OCF-II	500 × 10^3^	67.38
OCF-III	1 million	67.53

**Table 4:** Physical properties of the 500 mol/m^3^ NaCl solution at 25 °C.

Property	Value	Units
Density, *ρ*	1017	kg m^−3^
Viscosity, *µ*	0.931 × 10^−3^	N s m^−2^
Salt diffusivity, *D*	1.47 × 10^−9^	m^2^ s^−1^
Schmidt number, *(µ/ρ)/D*	622	-

**Table 5:** Grid dependence results (CFD).

FV CFD Mesh	No. Finite Volumes (*P/H* = 8, Re*_τ_* = 5.2, *γ* = 0°)	Darcy Friction Coefficient	Sherwood Number (Upper Wall)	Sherwood Number (Lower Wall)
OCF-A	2.252 × 10^6^	10.985	5.685	9.122
OCF-B	3.833 × 10^6^	11.062	5.519	8.771
OCF-C	7.502 × 10^6^	11.117	5.491	8.596

## Supplementary Materials

Supplementary materials can be found at https://www.mdpi.com/1422-0067/20/8/1840/s1. Figure S1. Engineering Stress vs Strain curve of the anion exchange membrane tested immersed in water. The linear elastic region is marked by a dashed line. The stress and strain at break are also highlighted. Figure S2. Experimental setup. (a) Overall layout; (b) Bulge test cell and laser head; (c) Detail of the Plexiglas plates making up the bulge test cell.

## Abbreviations

CFD	Computational Fluid Dynamics
ED	ElectroDialysis
FE	Finite Element
FV	Finite Volume
OCF	Overlapped Crossed Filaments
RED	Reverse ElectroDialysis
