# Supplementary material for: Membrane Deformation and Its Effects on Flow and Mass Transfer in the Electromembrane Processes
Source: Int J Mol Sci. 2019 Apr 13;20(8):1840. doi: 10.3390/ijms20081840 (PMC6515201; doi:10.3390/ijms20081840)
Supplement: Supplementary file 1 [file ijms-20-01840-s001.pdf]

International Journal of
Molecular Sciences

MDPI

1. Membranes Tested

The experimental tests described in this section were conducted on flat anion exchange membranes produced by FujiFilm Manufacturing Europe B.V. with the name "Type 10". According to information provided by the manufacturer, Type 10 is a homogeneous reinforced membrane and is based on amide polymer as backbone. The membrane is produced by roll to roll UV curing and it doesn't contain filler material.

2. Uniaxial Tensile Tests

Flat anion exchange membrane samples were conditioned for 24 h under tap water. The membrane was cut into rectangular strips of 30 cm (*L*) × 1.5 cm (*W*) and the wet membrane thickness, measured by a micrometer, was ~148±5 µm, as determined from different measurements conducted along the membrane length. Uniaxial tensile tests were first performed on samples immersed in water in a plastic bag. The uniaxial tensile testing machine, Zwick Roell Z010, equipped with a 10kN load cell, was used. Tests were run at a constant displacement rate of 30 mm/min following the ASTM D882-02 standard procedure. The gauge length was set at 15 cm and five specimens were tested. Experimental data in the form of the engineering stress-strain curve are shown in Fig. S1. The membrane shows a linear elastic region up to a stress value of ~2.3 MPa, with a Young's modulus of ~250 MPa. The stress and strain at break are ~13.9 MPa and ~0.125, respectively.

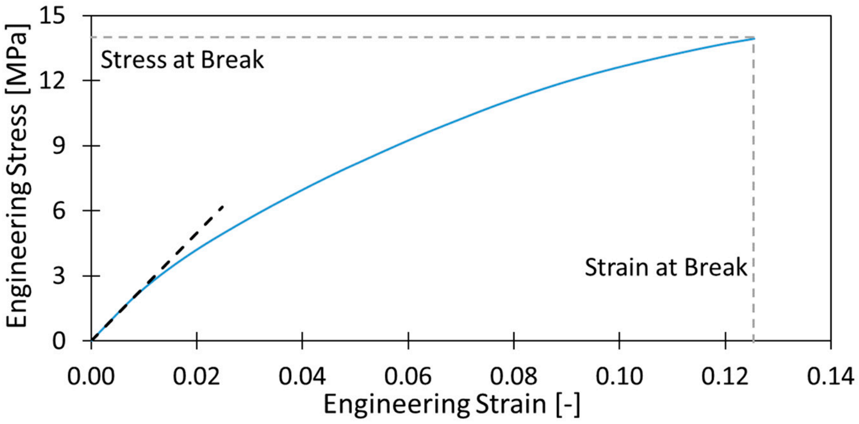

Figure S1. Engineering Stress vs Strain curve of the anion exchange membrane tested immersed in water. The linear elastic region is marked by a dashed line. The stress and strain at break are also highlighted.

3. Bulge Tests

In order to investigate the mechanical response of a flat membrane under uniformly distributed loads (hydrostatic head), a custom-designed bulge test equipment was built (Figure S2). Two square plexiglas® plates of 20 cm side and 2 cm thickness were used. Six circular holes with a diameter of 1.2 cm were drilled in one of the two plates (Plate 1) in order to allow water entrance and exit. The inlet and outlet manifolds were then installed into the holes. Moreover, a square hole of 10 cm side was milled in the other plate (Plate 2), to serve as a window allowing the membrane expansion. The membrane was conditioned for 24 h in tap water and then cut into square sheets of 20 cm side. A silicone gasket 1 mm thick creating the space for the liquid was interposed between the membrane sample and Plate 1. The two plates were clamped by 8 bolts and nuts. The bottom manifolds were connected to a water column. Once the channel was filled and air bubbles were carefully removed, a valve placed in the exit pipe was closed.

Membrane wrinkling was observed when no pre-stretching was applied. This is a common phenomenon, which happens for very thin sheets with negligible flexural stiffness if compressive residual stresses are present. Applying in-plane tensile forces (i.e., pre-stretching the membrane biaxially in its plane) would reduce this phenomenon. However, it would require a suitable equipment that was not available. Therefore, we preferred to slightly pre-stretch the membrane by applying an initial hydrostatic load of ~1 kPa (10 cm water column). The initial maximum displacement of the central point of the membrane was measured to be ~7.3 mm. The membrane was then pressurized by increasing the liquid height. The central membrane displacement was measured by a highly accurate laser scanner vibrometer (Polytech® PSV-400) and recorded at various levels of applied pressure increasing in 2 kPa steps up to 10 kPa. Tests were repeated five times for repeatability purposes. Corrected displacements were obtained by subtracting the initial displacement from the measured values.

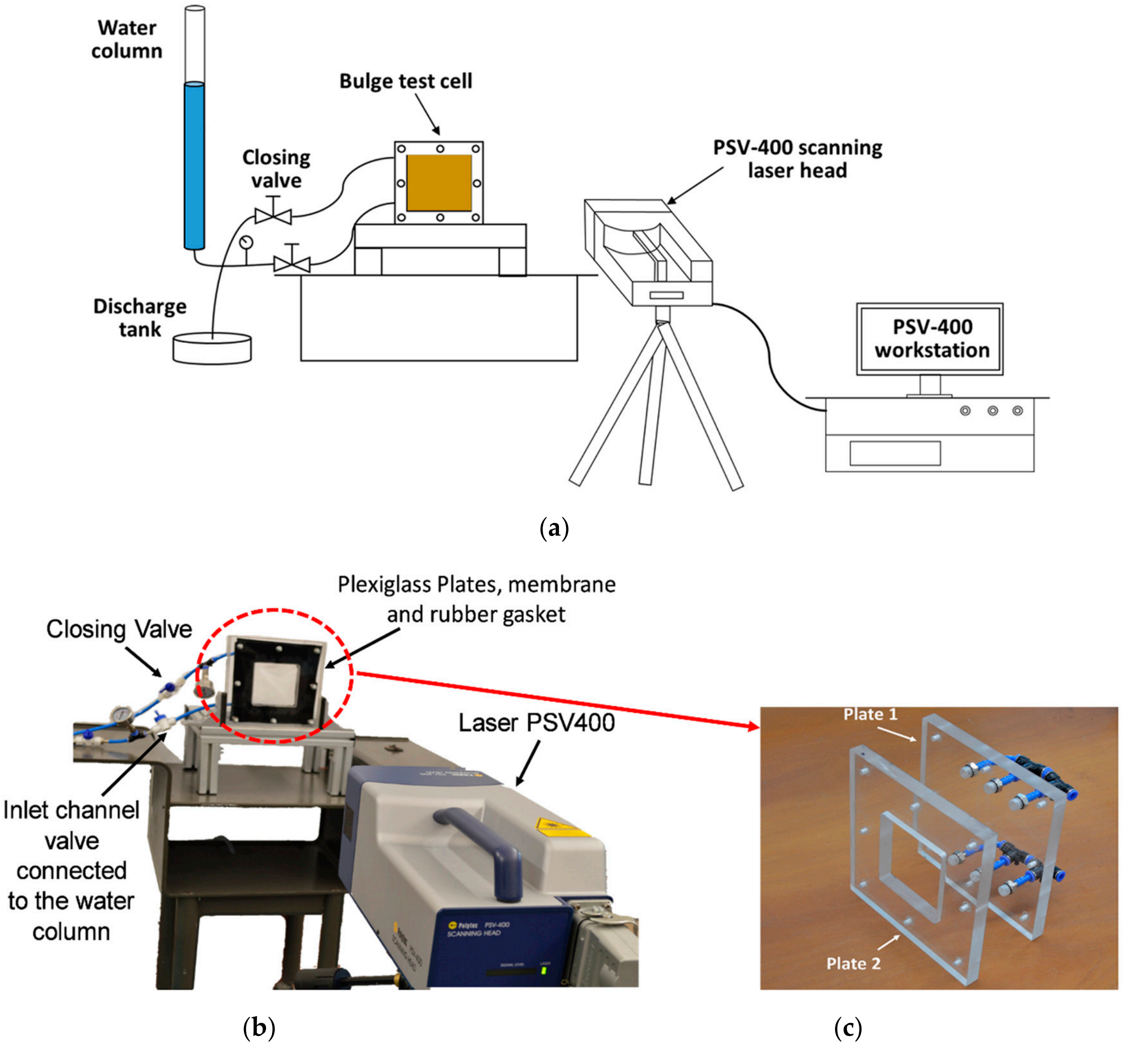

Figure S2. Experimental setup. (**a**) Overall layout; (**b**) Bulge test cell and laser head; (**c**) Detail of the Plexiglas plates making up the bulge test cell.

Membrane bulging was simulated by the FE model. Membrane mechanical properties and thickness, experimentally determined as discussed in the previous section (Young's modulus 250 MPa, thickness 148 μm), were provided as input to the model, taking into account the non-linear elastic behaviour. A Poisson ratio of 0.4 was considered, as suggested by the producer. As boundary conditions, all edges of the square membrane were clamped. The small deformation approximation was not used. First, the deformed stretched configuration (no-wrinkles) was numerically determined, knowing the maximum central displacement value. Then, a uniform pressure was applied to the pre-

stretched configuration. The comparison between the numerical results and the corrected measured displacements is shown in the paper (Figure 11a).

© 2019 by the authors. Licensee MDPI, Basel, Switzerland. This article is an open access article distributed under the terms and conditions of the Creative Commons Attribution (CC BY) license (http://creativecommons.org/licenses/by/4.0/).
